# 30-day and 1-year mortality after skeletal fractures: a register study of 295,713 fractures at different locations

**DOI:** 10.1080/17453674.2021.1959003

**Published:** 2021-07-26

**Authors:** Camilla Bergh, Michael Möller, Jan Ekelund, Helena Brisby

**Affiliations:** a Institute of Clinical Sciences, Sahlgrenska Academy, University of Gothenburg, Gothenburg; b Department of Orthopaedics, Sahlgrenska University Hospital, Gothenburg; c Centre of Registers Västra Götaland, Gothenburg, Sweden

## Abstract

Background and purpose — Few studies have reported the mortality rate after skeletal fractures involving different locations, within the same population. We analyzed the 30-day and 1-year mortality rates following different fractures.

Patients and methods — We included 295,713 fractures encountered in patients 16–108 years of age, registered in the Swedish Fracture Register (SFR) from 2012 to 2018. Mortality rates were obtained by linkage of the SFR to the Swedish Tax Agency population register. The standardized mortality ratios (SMR) at 30 days and 1 year were calculated for fractures in any location and for each of 27 fracture locations, using age- and sex-life tables from Statistics Sweden (www.scb.se).

Results — The overall SMR at 30 days was 6.8 (95% CI 6.7–7.0) and at 1 year 2.2 (CI 2.2–2.2). The SMR was > 2 for 19/27 and 13/27 of the fracture locations at 30 days and 1 year, respectively. Humerus, femur, and tibial diaphysis fractures were all associated with high SMR, at both 30 days and 1 year.

Interpretation — Patients sustaining a fracture had approximately a 7-fold increased mortality at 30 days and over 2-fold increased mortality at 1 year as compared with what would be expected in the general population. High mortality rates were seen for patients with axial skeletal and proximal extremity fractures, indicating frailty in these patient groups.

Compared with other medical conditions, the mortality rate after fractures has been considered to be low, and has not been frequently reported, with the exception of extensive literature on hip femur fractures; for review see Huette et al. (2020). For hip fractures, the importance of organizing care to decrease complications and mortality has been reported (von Friesendorff et al. 2016). Longer waiting time for surgery has reportedly been associated with increased mortality rates in some studies (Schnell et al. 2010, Pincus et al. 2017). A relationship between fractures in different locations and mortality rates can provide information on whether fractures in also other locations should be prioritized for treatment (Vestergaard et al. 2007a, Klop et al. 2017). Fracture types reported to be associated with increased mortality rates are vertebral fractures, distal radius fractures, diaphyseal, and distal femur fractures (Kado et al. 2003, Oyen et al. 2014, Larsen et al. 2020). There are, however, few reports comparing mortality rates for more than a few different fracture locations, within the same population. Hence, comparisons between mortality rates for different fracture locations are difficult.

To describe the change in mortality rate associated with a specific condition, the standardized mortality ratio (SMR) is commonly used (Vandenbroucke 1982). We investigated the 30-day and 1-year SMR for patients with fractures in various locations by using data from the Swedish Fracture Register (SFR).

## Patients and methods

### Data collection in the Swedish Fracture Register (SFR)

Data collection in the SFR began in 2011 and the data collection procedure has been described in detail by Wennergren et al. (2015). The number of hospitals attached to the SFR has gradually increased and at the end of 2020, 100% coverage was achieved with participation of all 54 departments treating fractures in Sweden. The completeness of fracture registrations in the SFR compared with the National Patient Register in 2018 was 70–95% for most participating departments.

All fractures, regardless of treatment (surgically or non-surgically), are prospectively registered in the SFR and classified according to the Müller AO/OTA classification system (Müller et al. 1990). Independent validation studies for different fracture locations regarding fracture classification have been performed (Juto et al. 2016, Wennergren et al. 2016, 2017, Knutsson et al. 2019, Morgonsköld et al. 2019). In addition to the classification of the fracture, the physicians/surgeons responsible for the fracture registration label each fracture according to the trauma mechanism as high, low, or undefined/unknown regarding energy type. The patient’s personal identification number allows for the monitoring of patients over time and enables accurate linkage to other national databases.

### Fracture classification and calculation of standardized mortality ratios

We included all fractures in patients aged 16 years and older, registered in the SFR between January 1, 2012 and December 31, 2018. Based on AO/OTA classification, fractures were divided into 27 anatomical regions (locations). Multiple fractures occurring at the same time, in the same patient, and in the same anatomical region, including bilateral fractures, were counted as 1 fracture for this analysis. Fractures occurring at the same time in different anatomical regions were counted once in each region. Subsequent fractures in a patient, regardless of anatomical region, were included as different entities in the analysis. Data on mortality for patients registered in the SFR were obtained by linkage of the SFR to the Swedish Tax Agency population register.

The SMR was calculated for all fracture locations together as well as for each of the 27 fracture locations by using the number of deaths among the fracture patients, divided by the expected number of deaths, based on the age- and sex-specific rates in the overall population and the size of the fracture population.

### Statistics

Mortality rates at 30 days and at 1 year were calculated as the number of patients who died divided by the total number of patients (for each fracture localization, and total, as appropriate) and expressed as a percentage. The SMR was calculated using mortality among patients registered in the SFR and the corresponding life tables for 2012–2018 retrieved from Statistics Sweden (www.scb.se). The life tables used report the 1-year mortality rates for each year of age and sex separately, for each of the relevant years. When calculating SMR for the 30-day period, this was done under the assumption that the expected number of deaths during 30 days would be 1/12th of that expected at 1 year based on the 1-year life tables. The SMR was calculated as the ratio between observed and expected mortality with 95% confidence interval (CI), according to the method by Vandenbroucke (1982). All calculations for tables and figures were performed using SAS (v9.4; SAS Institute, Cary, NC, USA).

### Ethics, funding, data sharing, and potential conflicts of interest

This study was approved by the Central Ethical Review Board, Gothenburg (ID 792-17). This research was supported by grants from the Swedish Research Council, Government Funding of Clinical Research within the National Health Service (ALF), from Västra Götaland ALFGBG722931, the Felix Neubergh Foundation, and the Gothenburg Medical Association, all in Sweden. The data that supports the findings of this study is available from the corresponding author on reasonable request. The authors declare that they have no competing interests.

## Results

### Baseline characteristics

During the study period 295,713 fractures were registered in the SFR (based on the anatomic regions definition, i.e., we analyzed 1 fracture from the same location for multiple fractures within the same location, at the same time of injury). These were sustained during 284,625 injury occasions, where 274,934 injury occasions involved a single location (97% of injuries), and 9,691 injury occasions involved more than 1 location (3.4%). For patients sustaining 2 or more fractures, the average number of fractures was 2.14. The cohort included 262,598 patients (59% women) (Tables 1 and 2).

**Table 1.** Overall descriptive data

Sex	Number of fractures	Age	
		mean (SD)	median (range)
Men	120,596	52 (23)	51 (16–107)
Women	175,117	66 (20)	69 (16–108)
All	295,713	60 (22)	64 (16–108)

**Table 2.** Fractures in all locations, fractures in another location at the same time, and a new fracture within a year. Values are count (%)

Fracture location	Number of fractures	Fracture in another location	New fracture within a year
All fractures	295,713	20,779 (7.0)	10,502 (3.6)
Spine	7,658	768 (10.0)	291 (3.8)
Pelvis	8,793	1,207 (13.7)	562 (6.4)
Acetabulum	1,718	374 (21.8)	71 (4.1)
Femur proximal	5,1355	1,874 (3.6)	2,620 (5.1)
Femoral diaphysis	2,786	300 (10.8)	130 (4.7)
Femur distal	2,476	309 (12.5)	123 (5.0)
Humerus proximal	23,572	2,009 (8.5)	998 (4.2)
Humeral diaphysis	3,267	368 (11.3)	174 (5.3)
Humerus distal	2,379	388 (16.3)	120 (5.0)
Distal radius	50,610	2,927 (5.8)	1,679 (3.3)
Tibia proximal	6,450	629 (9.8)	204 (3.2)
Tibia diaphysis	3,233	358 (11.1)	87 (2.7)
Tibia distal	2,283	274 (12.0)	56 (2.5)

High-energy trauma accounted for 8% of the fractures and was more commonly registered in men (15%) than in women (3.8%). For injuries involving more than 1 location the 30-day mortality was 2.3% (SMR 7.3, CI 6.4–8.3) and for single location injuries 2.0% (SMR 6.8, CI 6.6–7.0) for single location injuries. The corresponding numbers at 1 year were 7.9% (SMR 2.1, CI 2.0–2.3) and 7.8% (SMR 2.2, CI 2.2–2.2).

### Overall mortality rates

**Figure 1.**
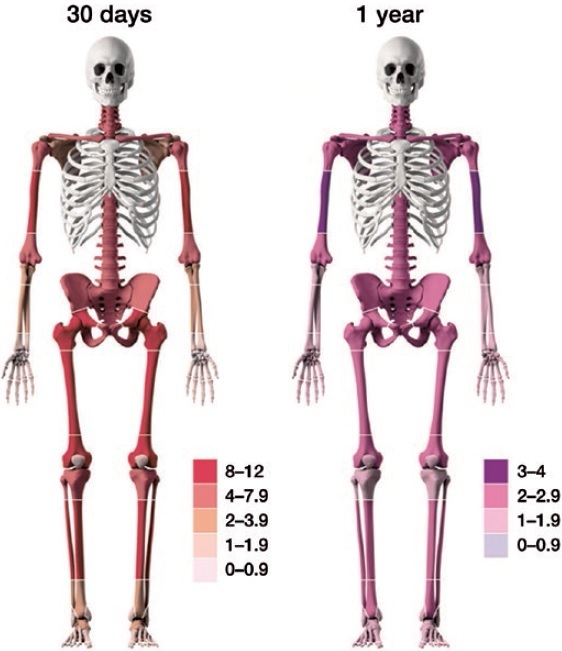
30-day (left) and 1-year (right) SMR in different body locations illustrated with color intensity, based on SMR figures. Cut-offs and SMR color codes are different for 30-day and 1-year SMR.

The overall mortality for fracture patients (any location) in the study population was markedly higher compared with expected mortality in the general population. The SMR was 6.8 at the 30-day time period and 2.2 for the 1-year time period (Table 3). The 30-day SMR in patients with fractures caused by high-energy trauma was 7.5 and 6.8 for low-energy trauma. Corresponding numbers at 1 year were 1.8 and 2.1 respectively (Table 3). An SMR > 2 was observed for 19/27 and 13/27 fracture locations at the 30-day and 1-year time points, respectively (Tables 4, 5, and 6). The SMR at 30 days and 1 year for the different fracture locations are illustrated in Figure 1.

**Table 3.** 30-day and 1-year mortality rate in patients sustaining a fracture at any location

						Proportion dead (%)		SMR (CI)	
						energy trauma		energy trauma	
Follow-up time	Number of fractures	Mean age years (SD)	Proportion dead (%)	Expected dead (%)	SMR (CI)	high	low	high	low
30-days	295,713	60 (22)	2.0	0.3	6.8 (6.6–7.0)	0.4	2.2	7.5 (6.1–9.0)	6.8 (6.6–6.9)
1-year	295,713	60 (22)	7.8	3.5	2.2 (2.2–2.2)	1.2	8.4	1.8 (1.6–2.0)	2.1 (2.1–2.2)

**Table 4.** Mortality rates at 30 days and 1 year for patients sustaining shoulder and upper extremity fractures

				30 days			1 year		
Fracture location	Number of fractures	Mean age years (SD)	High-energy (%)	Proportion dead (%)	Expected dead (%)	SMR (CI)	Proportion dead (%)	Expected dead (%)	SMR (CI)
Scapula	2,957	58 (19)	23.3	0.6	0.2	3.7 (2.2–5.6)	4.3	2.1	2.0 (1.7–2.4)
Clavicle	10,319	49 (22)	22.1	0.6	0.2	4.1 (3.1–5.1)	4.0	1.8	2.2 (2.0–2.4)
Humerus proximal	23,572	69 (16)	3.9	1.7	0.3	5.3 (4.8–5.9)	7.4	3.8	2.0 (1.9–2.0)
Humerus diaphysis	3,267	63 (21)	9.6	3.2	0.3	11.0 (9.0–13.0)	12.4	3.6	3.5 (3.1–3.8)
Humerus distal	2,379	66 (21)	8.0	2.4	0.3	7.0 (5.3–8.9)	9.7	4.2	2.3 (2.0–2.6)
Forearm proximal	11,701	51 (20)	7.5	0.4	0.1	3.2 (2.4–4.3)	2.3	1.4	1.6 (1.4–1.8)
Forearm diaphysis	2,204	50 (23)	23.0	0.4	0.2	2.9 (1.4–5.0)	3.5	1.9	1.8 (1.4–2.3)
Forearm distal (wrist)	50,610	61 (19)	5.3	0.4	0.2	1.9 (1.6–2.1)	2.8	2.5	1.1 (1.1–1.2)
Carpal	4,778	42 (20)	11.0	< 0.1	0.1	0.7 (0.1–2.0)	0.8	0.7	1.0 (0.7–1.4)
Metacarpal	16,821	41 (21)	8.3	0.1	0.1	1.0 (0.6–1.6)	1.4	1.0	1.4 (1.2–1.6)
Phalanx	17,267	46 (20)	12.2	0.1	0.1	1.0 (0.5–1.6)	1.2	1.0	1.2 (1.0–1.3)

**Table 5.** Mortality rates at 30 days and 1 year for patients sustaining fractures in the lower extremities

				30 days			1 year		
Fracture location	Number of fractures	Mean age years (SD)	High-energy (%)	Proportion dead (%)	Expected dead (%)	SMR (CI)	Proportion dead (%)	Expected dead (%)	SMR (CI)
Acetabulum	1,718	71 (19)	25.2	4.3	0.5	8.2 (6.4–10.1)	15.5	6.4	2.4 (2.1–2.7)
Femur proximal	51,355	81 (11)	1.4	7.5	0.8	10.0 (9.7–10.3)	24.6	9.2	2.7 (2.6–2.7)
Femur diaphysis	2,786	71 (22)	15.6	6.2	0.6	11.0 (9.4–12.8)	18.3	6.8	2.7 (2.5–2.9)
Femur distal	2,476	73 (19)	7.3	4.8	0.5	8.9 (7.4–10.6)	17.7	6.6	2.7 (2.4–2.9)
Patella	3,700	62 (20)	6.1	0.4	0.2	1.7 (0.9–2.6)	3.0	2.8	1.1 (0.9–1.3)
Tibia proximal	6,450	56 (20)	17.4	0.7	0.2	4.2 (3.0–5.5)	3.8	2.0	1.9 (1.7–2.1)
Tibia diaphysis	3,233	50 (21)	23.5	1.1	0.1	8.2 (5.7–11.1)	4.8	1.7	2.9 (2.5–3.4)
Tibia distal	2,283	51(21)	23.8	0.4	0.1	3.0 (1.4–5.2)	4.2	1.8	2.4 (1.9–2.9)
Ankle	32,975	55 (19)	5.5	0.3	0.1	1.9 (1.6–2.4)	2.2	1.6	1.4 (1.3–1.5)
Talus	960	39 (17)	38.8	0.1	< 0.1	3.2 (0.0–12.4)	0.4	0.4	1.0 (0.3–2.3)
Calcaneus	1,919	48 (18)	35.0	0.2	0.1	2.9 (0.8–6.5)	1.3	0.9	1.5 (1.0–2.2)
Midfoot	2,212	43 (18)	20.9	0.1	< 0.1	3.2 (0.6–7.7)	9.0	0.5	1.7 (1.0–2.6)
Metatarsal	12,475	48 (20)	5.8	0.1	0.1	0.8 (0.4–1.5)	1.4	1.1	1.3 (1.1–1.5)
Toe phalanx	8,845	46 (18)	6.5	0.1	0.1	1.6 (0.7–2.9)	0.8	0.7	1.1 (0.9–1.4)

**Table 6.** Mortality rates at 30 days and 1 year for patients sustaining spinal and pelvic fractures

				30 days			1 year		
Fracture location	Number of fractures	Mean age years (SD)	High-energy (%)	Proportion dead (%)	Expected dead (%)	SMR (CI)	Proportion dead (%)	Expected dead (%)	SMR (CI)
Spine	7,658	64 (22)	25.0	2.4	0.3	6.9 (5.9–8.0)	10.1	4.2	2.4 (2.3–2.6)
Pelvis	8,793	75 (19)	10.9	3.8	0.6	6.1 (5.4–6.8)	16.7	7.7	2.2 (2.1–2.3)

**Figure 2.**
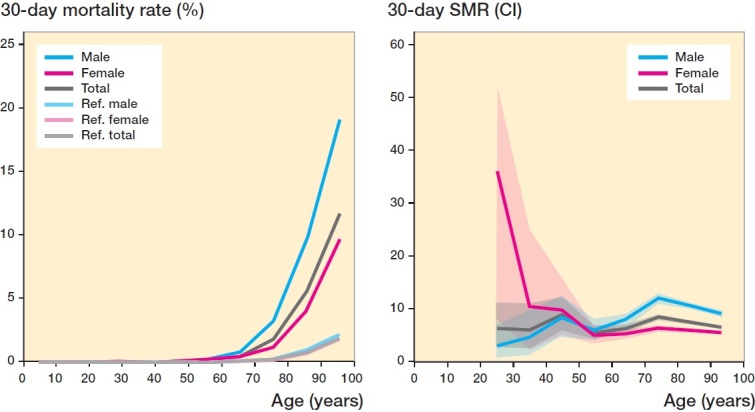
30-day mortality rate of all fractures for different ages (from 16 years), and sorted by sex in percentages (left). Normal population reference values included. 30-day SMR with 95% CI for all fractures for different ages (from 16 years) and sorted by sex (right). There were no observed deaths within 30 days for individuals below 24 years.

**Figure 3.**
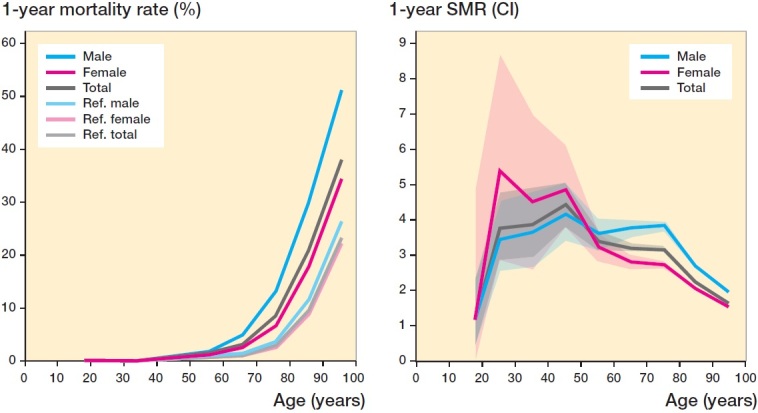
1-year mortality rate for all fractures for different ages (from 16 years) and sorted by sex in percentages (left). Normal population reference values included. 1-year SMR with 95% CI for all fractures for different ages (from 16 years) and sorted by sex (right).

Overall, mortality rates as well as SMRs were higher for men aged ≥ 60 years, compared with women, at both the 30-day and at 1-year time points (Figures 2 and 3). The large CI due to small absolute numbers of fractures seen for patients of younger age makes comparisons between sexes difficult in these age groups.

### Mortality rates at 30-day and 1-year for upper extremity fractures (including shoulder/scapula)

For the 145,875 fractures that the patients sustained in the upper extremities, the mortality rate was 0.4% (n = 519) at 30 days and 3.9% (n = 5,704) at 1 year. High mortality rates at 30 days were seen for humeral diaphysis (12%) and distal humerus fractures (7.9%), with SMR of about 11 and 7, respectively. At the 1-year time point, SMR for humeral diaphysis and distal humerus fractures was 3.5 and 2.3, respectively. Fractures in the shoulder region and of the proximal part of the upper extremity also had SMRs ≥ 2 at both time points (Table 4, Figure 2).

### Mortality rates at 30 days and 1 year for lower extremity fractures (including acetabulum)

For the 133,387 fractures in the lower extremities, the mortality rate was 3.6% (n = 4,826) at 30 days and 15% (n = 20,139) at 1 year. Almost all lower extremity fracture types revealed an SMR of 3 or above at 30 days. Fractures of the femur (all 3 locations) had SMR at 30 days of ≥ 8. At the 1-year time point, all femur fracture locations and tibia diaphysis fractures were associated with an SMR ≥ 2. (Table 5, Figure 1).

### Mortality rates at 30 days and 1 year for spine and pelvis fractures

Fractures of the spine and pelvis were associated with an SMR of roughly 6 at the 30-day time point and 2 at the 1-year time point (Table 6, Figure 1). 

## Discussion

In this national register study, we found that fractures of most locations had an increased mortality rate. For all locations a 7-fold higher mortality at 30 days and 2-fold higher mortality at 1 year were seen, when compared with expected mortality in the general population with the same age and sex distribution. The mortality rates varied largely across different fracture locations. Proximal extremity fractures of both the upper and lower extremities, as well as vertebrae and pelvic fractures, were all associated with high SMR.

The mortality rate is often reported for other acute medical conditions, e.g., myocardial infarction and stroke. The mortality rate within the first month was reported to be 25% after stroke and approximately 24% after myocardial infarction in Sweden in 2019 (National Board of Health and Welfare, Sweden 2020). The corresponding 30-day mortality rates in our study for proximal and diaphyseal femur fractures were 8% and 6%, respectively. The 1-year mortality rate after a myocardial infarction in 2019 in Sweden was approximately 33% (National Board of Health and Welfare, Sweden 2020), which is comparable to 25% for hip fractures and 18% for femoral diaphysis fractures. Most fracture locations in the proximal and diaphyseal parts of the long bones, vertebrae, and pelvis were associated with an SMR > 2 at 1 year.

An increase in mortality after lower extremity fractures has been previously reported (Somersalo et al. 2016, Huette et al. 2020), confirmed in our study by high SMR for patients with femur fractures, acetabulum fractures, and tibial shaft fractures. Hip fractures are among the most studied and common fractures. Proximal femur fractures had the highest mortality rate among all fracture locations; 25% at 1 year with a corresponding SMR of 2.7, which is in accordance with previous studies (Vestergaard et al. 2007b, Gundel et al. 2020). For comparison, distal femur fractures had a 30-day mortality rate of 4.8%, which is similar to the 5% mortality reported by Larsen et al. (2020) and 6.3% by Wolf et al. (2021). The 18% 1-year mortality for distal femur fracture patients in our study was lower than the 25% for proximal femur fractures, with a similar SMR of 2.7.

In the upper extremities, the diaphyseal and distal humerus fractures were associated with an SMR of 11 and 7.0, respectively, at 30 days, and of 3.5 and 2.3, respectively, at 1 year. Proximal humerus fractures were associated with an SMR of 5.3 at 30 days and 2.0 at 1 year. These results are in accordance with previous studies on proximal humerus fractures (Bercik et al. 2013, Wilson et al. 2014). A higher mortality rate was also seen after scapular and clavicular fractures at 1 year, both locations demonstrating an SMR of 2 or higher. Distal fractures in the upper and lower extremities were associated with only minor increases in mortality rates. The second most common fracture, after hip fracture, in our study was wrist fracture. A similar or slightly lower mortality rate of 2.5% at 1 year was seen for this location compared with the previously reported 3–3.6% (Endres et al. 2006, Oyen et al. 2014) and associated with a low SMR (1.1).

The mortality rate at 1 year after non-traumatic vertebral fractures was approximately 10%, which is lower than the previously reported 12–46% (Lau et al. 2008, Harris et al. 2010, Waterloo et al. 2012). Vertebral fractures in the elderly resulting from low-energy injuries are not commonly diagnosed at accident and emergency departments but are more likely to be diagnosed and treated in primary health care. This might have influenced the mortality seen in our study, which is based on fracture register data obtained from emergency departments.

The 30-day mortality rate is more likely to be influenced by factors directly linked to the fractures sustained than the 1-year mortality which may, to a larger extent, reflect the influence of comorbidities. We observed that the mortality rate from a fracture, compared with what would be expected, was higher at 30 days than at 1 year. It was beyond our scope to analyze the influence of comorbidities and the death cause. The mortality rate was, as expected, observed to be related to age, in accordance with previous reports of specific fracture locations such as the humerus (Ravindrarajah et al. 2018, Bergdahl et al. 2020) and the femur (Ravindrarajah et al. 2018, Wolf et al. 2021).

The 30-day and 1-year mortality in men over the age of 60 years was higher than in women. This is in agreement with previous studies where men with comorbidities or with low bone mineral density have been reported to have an increased mortality risk when sustaining frailty fractures (Bliuc et al. 2009, Cook et al. 2017). For a frail person, the fracture is an event, often in a multifactorial chain that increases the risk of death (Johnell et al. 2004, Huette et al. 2020). The independent role of the fracture versus other factors such as comorbidities has been reported to be uncertain in previous studies (Vestergaard et al. 2007b, Teng et al. 2008). High mortality in osteoporotic fractures has, though, been relatively well described (Bliuc et al. 2009, Alarkawi et al. 2020) and there is an ongoing discussion on possible mortality reduction with osteoporosis treatment (Bliuc et al. 2019). Further, in a paper from Borgen et al. (2019) patients with fractures of the axial skeletal and proximal extremities had a lower bone mineral density. This is the same pattern observed and visualized in our Figure 1.

Multiple fractures on the same occasion have been suggested to signal an increased risk of mortality (Sujic et al. 2020). In our study the mortality rate as well as SMR at 30 days was slightly higher for multiple fractures than for single location fractures and no differences were seen at 1 year.

The strength of our study is the use of structured data, collected within a national register and directly linked to the Swedish Tax Agency population register, providing the possibility to compare different fracture locations within a population.

The major limitations of our study are that data on comorbidities and cause of death was not available. It should be noted that direct comparison of SMRs between fracture locations (or between populations) is difficult due to potential differences in the distribution of standardizing variables. Future studies, preferably with linkage to other national health databases, may provide an opportunity to directly compare the mortality risk between fracture locations while controlling for standardizing variables and other potential confounders. Another limitation is that only lifetables for mortality per year were available, which is why seasonal variations of mortality and fracture incidence may have caused an over- or underestimation of the 30-day SMR figures. Another source of potential bias in the SMR calculations is the low completeness of registrations from some hospitals, which may influence the registrations of some fracture types more than others

In conclusion, patients who sustain a fracture had a marked increase in mortality rate at both 30 days and 1 year, about 7-fold and over 2-fold, respectively, when compared with the expected mortality rate of the Swedish population assuming the same age and sex distribution as in the study population. High SMR was seen for proximal fractures of both upper and lower extremities, but also for vertebrae, pelvic, and acetabular fractures. It is, however, important to emphasize that the size of SMR following specific fractures may be due to many factors: the fracture location, the treatment, the distribution of standardizing variables in the patient population, and other confounding factors.
